# Necrotizing enterocolitis intestinal barrier function protection by antenatal dexamethasone and surfactant-D in a rat model

**DOI:** 10.1038/s41390-020-01334-0

**Published:** 2021-01-19

**Authors:** Lei Lu, Jing Lu, Yueyue Yu, Erika Claud

**Affiliations:** Department of Pediatrics, Pritzker School of Medicine, The University of Chicago, Chicago, IL 60637 USA

## Abstract

**Background:**

Necrotizing enterocolitis (NEC) is the most common gastrointestinal disorder in premature neonates. Possible therapeutic approaches are centered on promoting maturation of the gastrointestinal mucosal barrier. Studies have demonstrated that antenatal administration of corticosteroids can decrease NEC incidence and mortality.

**Methods:**

Pregnant rat dams were administered dexamethasone 48 h prior to delivery. The pups were subjected to an experimental NEC-like injury protocol. Ileal tissues and sera were collected and evaluated for inflammatory cytokines, gut permeability and expressions and localizations of tight junction proteins, and surfactant protein-D by immunohistochemistry/immunofluorescent staining. Intestinal epithelial cells (IEC-6) were pretreated with SP-D to examine the effect of SP-D on tight junction protein expressions when challenged with platelet-activating factor and lipopolysaccharide to model proinflammatory insults.

**Results:**

Antenatal dexamethasone reduced systemic inflammation, preserved intestinal barrier integrity, and stimulated SP-D expression on the intestinal mucosal surface in pups exposed to NEC-like injury. Pretreatment of SP-D blocked platelet-activating factor/lipopolysaccharide-induced tight junction disruption in IEC-6 cells in vitro.

**Conclusions:**

Antenatal dexamethasone preserves the development of intestinal mucosal barrier integrity and reduces incidence and morbidity from an experimental NEC-like injury model. Dexamethasone upregulation of intestinal SP-D-protective effects on tight junction proteins.

**Impact:**

- Antenatal administration of dexamethasone can function in concert with intestinal surfactant protein-D to decrease systemic inflammatory responses, and protect intestinal barrier integrity in a neonatal rat model of NEC.
- A novel role of intestinal SP-D in preserving tight junction protein structures under inflammatory conditions.
- We describe the intestinal SP-D—an overlooked role of antenatal dexamethasone in neonatal NEC?

## Introduction

Necrotizing enterocolitis (NEC) is the most frequent gastrointestinal emergency of preterm infants, representing a major cause of morbidity and death in neonates.^1–3^ This disease is characterized by massive epithelial necrosis, gut barrier dysfunction, and improper mucosal defense development.^4–7^ Prematurity remains the most consistent risk factor for developing NEC.

Glucocorticoids (GCs) are suggested to promote the maturation of many vital fetal organs, for example, have been used to induce maturation of fetal lungs, and are associated with subsequent improved postnatal survival.^8^ In addition to the decrease in the incidence of respiratory distress syndrome (RDS), prenatal GC therapy has also been associated with decreased systemic infections, and decreased the incidence of other prematurity-associated diseases, such as intraventricular hemorrhage, patent ductus arteriosus, and NEC in infants born preterm.^8–12^ However, the mechanism by which corticosteroids are protective in NEC remains largely unknown.

Animal studies have demonstrated that prenatal cortisone acetate administration reduces both incidence and morbidity in a rat model of NEC partially due to increased maturation of the intestinal mucosal barrier evidenced by decreased bacteria translocation, reduced uptake of macromolecules, and lower intestinal permeability.^13,14^ In fetal lungs, GCs stimulate the production of surfactant-associated proteins.^15^ Surfactant protein-A (SP-A) and surfactant protein-D (SP-D) are established as essential components of the innate immune system for protecting against pathogens.^16^ In the gastrointestinal tract, SP-A and SP-D are reported to express in the epithelial lining of the small and the large intestine and surfactant proteins have been shown in in vivo and in vitro models to contribute to protection against NEC.^17–19^ Tight junctions (TJs) are known to function as barriers in the intestinal tract. Recently, TJs have emerged as dynamic heteromeric signaling complexes acting not only to regulate its assembly and function, but also to coordinate signals regulating gene expression and subsequent cellular responses, like cell proliferation and differentiation.^20^ Zonula occludens-1 (ZO-1) can interact with different components of tight and adherens junctions and to play a modulatory role during the formation of TJs. ZO-1 also regulates gene expression, cell proliferation, and epithelial morphogenesis by its interaction with the heat-shock protein Apg-2. Apg-2 regulates junction assembly and is an important regulator of epithelial differentiation.^21,22^ In this study, we demonstrate that prenatal GC is effective in protecting offspring from NEC in part by regulating SP-D and APG2, which play important roles in TJ development and function.

## Materials and methods

### Animals

This study was carried out in strict accordance with the recommendations in the Guide for the Care and Use of Laboratory Animals of the National Institutes of Health. All animal work was conducted under the animal protocol no. 71557 and was approved by the University of Chicago Institutional Animal Care and Use Committee (IACUC). Time-dated pregnant Sprague–Dawley dams (from Harlan) were housed and allowed ad libitum access to food and water in the animal care facilities of the University of Chicago. Some of the dams were administered a single dose of dexamethasone (40 ng/g body weight intraperitoneally) 48 h prior to Cesarean section.

### Neonatal rat experimental intestinal injury model

Neonatal rats from time-dated pregnant Sprague–Dawley dams were delivered by cesarean section at E20 following isoflurane anesthesia. Pups were fed with Esbilac puppy formula (Pet-Ag, Inc., Hampshire, IL) every 3 h via an orogastric feeding catheter and colonized with 10^7^ colony-forming unit (CFU) of *Serratia marcescens*, *Klebsiella pneumoniae*, and *Streptococci viridans* in 100 µl formula once daily. Pups were stressed with 5% O_2_ + 95% N_2_ for 10 min after feeding three times a day. The feeding volume began at 0.1 ml and was increased incrementally up to 0.25 ml. If a rat pup showed illness during the course of the study, the animal was humanely euthanized and death was not used as an endpoint. The remaining viable animals were weighed and euthanized at the end of the experiment on day 5. The terminal ileal segments (~4 cm in length) and sera were collected for histological and biochemical studies.

### NEC-like injury evaluation

The rat intestine was evaluated by gross inspection for the presence of necrosis and then formalin-fixed, paraffin-embedded, microtome-sectioned, and stained with hematoxylin and eosin for histological evaluation. Histological findings were scored by a blinded pathologist as follows: 0, no histological damage; 1, mild with epithelial sloughing; 2, moderate with mid villous necrosis present as moderate separation of the submucosa and/or lamina propria and/or edema in the submucosa and muscular layers; 3, severe with total villous necrosis; and 4, transmural necrosis. Scores ≥2 were defined as NEC-like intestinal injury.^23^

### Cytokine assay

The multiplex analysis was performed using a kit containing a panel of rat cytokines/chemokines, which utilizes Luminex® xMAP® technology with magnetic beads, according to the manufacturer’s instructions (Bio-Rad, Hercules, CA). Experiments were performed in triplicate. The kit enables simultaneous analysis of 12 cytokines, chemokines, and interleukins (ILs) in a 25 µl (2× diluted) serum sample.

### In vivo intestinal barrier function assay

To investigate the intestinal lumen-to-blood permeability in the newborn rat, fluorescein isothiocyanate (FITC)-labeled dextran molecules were fed to neonatal rats and the plasma was sampled to assess mucosal permeability as described previously.^24^ Based on our previous study 10-kDa FITC-dextran administration produced consistent results in neonatal rats. Briefly, all surviving rat pups (injury score “0”) at the end of the experiments on day 5 were fed with 10-kDa FITC-dextran (40 mg/100 g body weight) via an orogastric tube. Two hours later, whole blood was collected and serum was used for measurement of fluorescence intensity. High concentrations of FITC-dextran in the serum indicate poorer barrier function.

### Immunohistochemistry and immunofluorescence

Ileal tissues from the surviving rat pups were formalin-fixed, paraffin-embedded, processed, and cut into 5-μm sections. For immunohistochemistry, 5-μm paraffin sections were deparaffined, rehydrated, and then endogenous peroxidase was quenched with H_2_O_2_ (3% in methanol). Antigen retrieval was performed by microwave in a citrate buffer. Primary antibodies or isotype controls were added to the sections, followed by incubation with the biotinylated secondary antibody and finally detected using DAB solution (ABC Detection Kit, Vector Laboratories). Sections were counterstained with hematoxylin (Dako, Carpinteria, CA) and scanned.

For immunofluorescence, rehydrated tissue sections were incubated with blocking solution (5% goat serum in phosphate-buffered saline) for 1 h. The tissue sections were incubated with 100 μl of the respective primary antibody solution overnight at 4 °C and then the sections were incubated with the respective fluorophore-conjugated secondary antibodies and counterstained with DAPI (4′,6-diamidino-2-phenylindole)-antifade mounting medium (Invitrogen Inc., Carlsbad, CA). Imaging was performed at the University of Chicago Integrated Light Microscopy Facility. Images were captured with a Leica TCS SP8 laser scanning confocal microscope (Leica Microsystems, Inc., Buffalo Grove, IL). Imaging processing and analysis were obtained using ImageJ (National Institutes of Health, Bethesda, MD, http://imagej.nih.gov/ij/).

### Real-time reverse transcriptase-polymerase chain reaction (RT-PCR)

Total RNA was extracted from frozen ileal samples using the RNA Easy Mini Kit (Qiagen GmbH, Hilden, Germany) according to the manufacturer’s instructions. Subsequently, complementary DNAs (cDNAs) were synthesized using First Strand cDNA Synthesis Kit (Qiagen). A portion of the RT products (equivalent to 10 ng RNA) was used directly for real-time PCR. Real-time PCR assays were performed on cDNA samples in 384-well optical plates on a 7900HT ABI Prism Sequence Detection System (Applied Biosystems, Foster City, CA).

### Cell cultures

Rat intestinal epithelial cells (IEC-6) were obtained from the American Type Culture Collection (ATCC, Rockville, MD) and routinely maintained per ATCC recommendation in Dulbecco’s modified Eagle’s medium containing 5% fetal calf serum, 100 U insulin, 50 mg/L penicillin, and 50 mg/L streptomycin. All experiments were done on cells between passages 22 and 30. Upon reaching 70–80% confluency, IEC-6 cells were incubated with platelet-activating factor (PAF, 10 ng/ml) for 20 h and then treated with lipopolysaccharide (LPS, 100 ng/ml). In some experiments, IEC-6 cells were preincubated in IEC-6 media containing 20 ng/ml of SP-D for 1 h prior to further treatment with PAF/LPS. All PAF/LPS stimulation experiments were done in serum-free Hank’s balanced salt solution supplemented with 0.25% bovine albumin.

### Western blot analysis

IEC-6 cells were lysed in RIPA buffer containing protease inhibitors. Protein concentrations were determined using the Pierce TM BCA protein assay (Thermo Scientific, Rockford, IL); 25 μg of each preparation was denatured in standard Laemmli buffer, separated on a 12% sodium dodecyl sulfate-polyacrylamide gel, transferred onto PVDF membrane (Bio-Rad), and probed for antigen detection.

### Co-immunoprecipitation (co-IP)

IEC-6 cells were lysed in 1 ml of lysis buffer (1% NP40, 20 mM Tris, 150 mM NaCl, and 2 mM EDTA [Sigma-Aldrich], pH 7.4). 1× Complete protease inhibitor cocktail (Roche Applied Sciences). One microgram of the appropriate IP antibody was incubated with 50 μl of packed protein G Sepharose (Sigma-Aldrich) in 1 ml PBS incubated overnight at 4 °C under rotation. The pellets were collected by centrifugation and washed four times with PBS. Then a total of 500 μg of protein extract in 1 ml lysis buffer was added to the antibody-conjugated protein G Sepharose and incubated for 1 h under rotation. The pellets were collected by centrifugation and washed four times with lysis buffer for 5 min. The pellets were resuspended in 50 μl of 2× Laemmli buffer (Bio-Rad) and ready for western blot analysis using antibodies directed against ZO-1, APG2, or heat-shocking protein 70 (HSP70).

### Antibodies and reagents

Mouse monoclonal antibody occludin (OCLN) and ZO-1 were obtained from Invitrogen. SP-D antibody and secondary Alexa Fluor-488-goat anti-rabbit immunoglobulin G (IgG) and Alexa Fluor-594-goat anti-mouse IgG were purchased from Thermo Fisher Scientific (Rockford, IL). Antibodies against APG2, total, and phosphorylated MLC2 were purchased from Cell Signaling Technology (Danvers, MA). PAF, SP-A, and SP-D were purchased from R&D Systems (Minneapolis, MN). Dexamethasone, LPS, and other chemicals were obtained from Sigma (St. Louis, MO).

### Statistics

All data are presented as means ± SEM. Statistical analysis was performed using the *χ*^2^ test for NEC-like intestinal injury incidence and Student’s *t* test for paired data with GraphPad Prism software (San Diego, CA). Differences were considered to be significant with *p* values <0.05.

## Results

### Antenatal dexamethasone protects against NEC-like injury incidence and severity and decreased serum inflammatory cytokine levels in response to experimental NEC challenge in neonatal rats

**Fig. 1:**
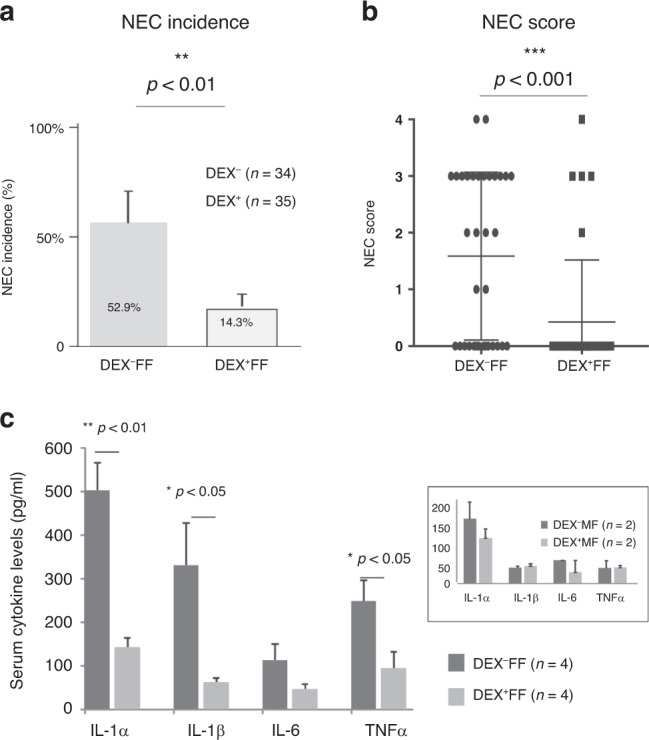
Antenatal dexamethasone decreases NEC incidence, severity, and serum inflammatory cytokine levels. The incidence of NEC (**a**), histologic scores of NEC from ileal tissue (**b**) in DEX^−^FF (*n* = 34) and DEX^+^FF (*n* = 35) groups. The histologic scores: 0, no histological damage; 1, mild with epithelial sloughing; 2, moderate with mid villous necrosis present as moderate separation of the submucosa and/or lamina propria and/or edema in the submucosa and muscular layers; 3, severe with total villous necrosis; and 4, transmural necrosis. **c** Serum levels of inflammatory cytokines IL-1α, IL-1β, IL-6, and TNF-α were quantified using a multiplex immunoassay in DEX^−^MF and DEX^+^MF pups (*n* = 2 per group) (inset), and in DEX^−^FF (*n* = 4) and DEX^+^FF (*n* = 4). The results are presented as mean ± SEM. Student’s *t* test was used to detect differences between the groups. Bars denote a significant difference between experimental groups (*p < 0.05, **p < 0.01, and ***p < 0.001).

In this study, the severity of intestinal injury (histology score) and the incidence of NEC were determined by a scoring system as described in “Methods.” In all experimental NEC animals, an injury score ≥2 was classified as NEC-like injury. Antenatal dexamethasone significantly reduced the incidence of NEC (from 52.9 to 14.3%, *p* < 0.01) and the severity of the injury (the mean histology score from 1.67 to 0.4, *p* < 0.001) (Fig. 1a, b). Both dam-fed (DEX^−^MF) and DEX-dam-fed (DEX^+^MF) pups exhibited no NEC-like injury. Thus, antenatal exposure to dexamethasone-protected neonatal rats from NEC-like injury. Previous studies have shown that tumor necrosis factor-α (TNF-α), IL-1β, and IL-6 are proinflammatory cytokines increased in human NEC patients and in animal models of NEC (reviewed in ref. ^25^). Moreover, IL-1α levels are highly correlated to intestinal inflammation, edema, and necrosis.^26^ In this study, we examined levels of inflammatory cytokines in the sera of neonatal rats in response to NEC. As shown in Fig. 1c, antenatal dexamethasone treatment significantly lowered systemic IL-1α, IL-1β, and TNF-α levels in the neonatal rats exposed to experimental NEC stress. Both DEX^−^MF and DEX^+^MF pups exhibited lower levels of systemic cytokines compared to the experimental NEC pups (Fig. 1c, inset). Dexamethasone decreases mucosal permeability and preserves the expression of MUC2 proteins and TJ proteins in the ileum during the course of experimental NEC challenge in the ileal surface and crypts during the course of experimental NEC challenge.

**Fig. 2:**
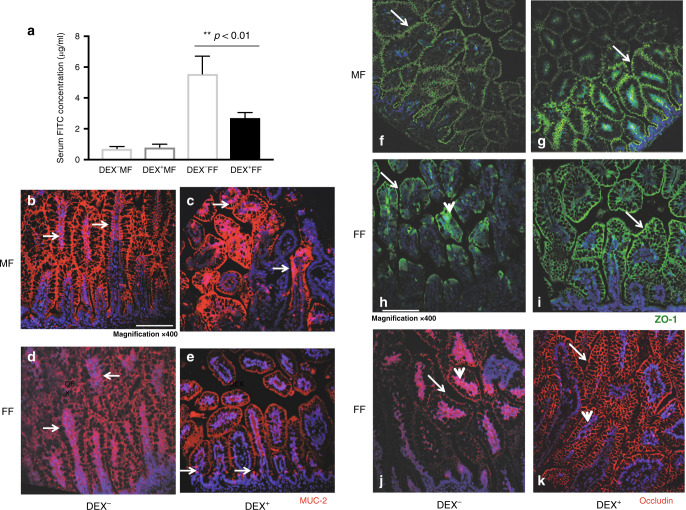
Dexamethasone decreases mucosal permeability and preserves the expression of MUC2 proteins and tight junction proteins in the ileum during the course of the experimental NEC challenge. **a** Serum levels of FITC-dextran in DEX^−^MF (*n* = 4), DEX^+^MF (*n* = 5), DEX^−^FF (*n* = 7), and DEX^+^FF (*n* = 6) pups were measured and presented as mean ± SEM. Student’s *t* test was used and **p < 0.01. Representative images of immunofluorescence detection of MUC2 molecules (red) in DEX^−^MF (**b**), DEX^+^MF (**c**), DEX^−^FF (**d**), and DEX^+^FF (**e**) apical surface or crypt MUC2 expressions were indicated by white arrows. Representative images of immunofluorescence detection of ZO-1 in DEX^−^MF (**e**), DEX^+^MF (**f**), DEX^−^FF (**g**), and DEX^+^FF (**h**) rat ileum. DEX^−^FF pups present a disruption of apical tight junction structure and intracellular translocation of ZO-1 and are indicated by block arrows. DEX^+^FF pups (**i**) exhibit significantly higher surface expression and less intracellular accumulation of ZO-1. **j** Representative images of immunofluorescence detection of OCLN molecules (red) in DEX^−^FF rat ileum. Intracellular translocation of OCLN molecules and disruption of apical tight junction structure is indicated by block arrows. **k** DEX^+^FF pups had significantly higher surface OCLN expression, and preservation of TJ structures (*n* = 3 per group are shown, bar: 100 μM, magnification ×400).

Maintenance of the intestinal epithelial barrier is an essential function of intestinal epithelial cells (IECs). Intestinal barrier permeability may be a prognostic marker for disease pathophysiology. The TJ proteins, ZO-1 and OCLN, are crucial components of epithelial barrier integrity. We assessed the effects of antenatal dexamethasone on intestinal mucosal permeability under the experimental NEC challenge in the neonatal rats. As shown in Fig. 2a, there were significantly lower serum levels of FITC-dextran in antenatal dexamethasone-exposed experimental NEC pups (*p* < 0.01; Fig. 2a) compared with the untreated NEC pups. DEX^−^MF and DEX^+^MF pups exhibited minimal serum levels of FITC. The mucus layer in the intestine affects several aspects of intestinal biology, encompassing physical protection, chemical protection, immunomodulation, and growth, thus playing a role in maintaining mucosal homeostasis of the immature intestinal barrier. As shown in Fig. 2, compared to DEX^−^MF pups, exposure enhanced baseline MUC2 expression in the DEX^+^MF pups ileal mucosa (Fig. 2b, c). Furthermore, when exposed to experimental NEC challenge, DEX^+^FF pups exhibited morphologically better mucosal surface lining and strong MUC2 expression in the crypts compared to DEX^−^FF pups (Fig. 2d, e).

Next, we evaluated TJ structure integrity during the experimental NEC challenge in the pups. As shown in Fig. 2f, g, both DEX^−^MF and DEX^+^MF pups exhibited comparable well-formed surface ZO-1 localization in the ileal tissues. However, there was disruption of barrier integrity represented by the prominent intracellular presence of TJ protein ZO-1 in experimental NEC ileal tissues (Fig. 2h, white arrow). In contrast, antenatal dexamethasone prevented the loss of surface ZO-1 (Fig. 2i). A similar disruption of TJ protein on the ileal apical surface and prominent intracellular translocation of OCLN is shown in Fig. 2j as indicated by the block arrow. Antenatal dexamethasone-protected neonatal ileal integrity from experimental NEC-induced barrier disruption (Fig. 2k, white arrow). These results provide a cellular basis for the preservation of mucosal barrier integrity in antenatal dexamethasone-treated neonatal rat ileum.

### Antenatal dexamethasone affects ileal mucosal surface expression of SP-D but not SP-A in the rat model of experimental NEC-like injury

**Fig. 3:**
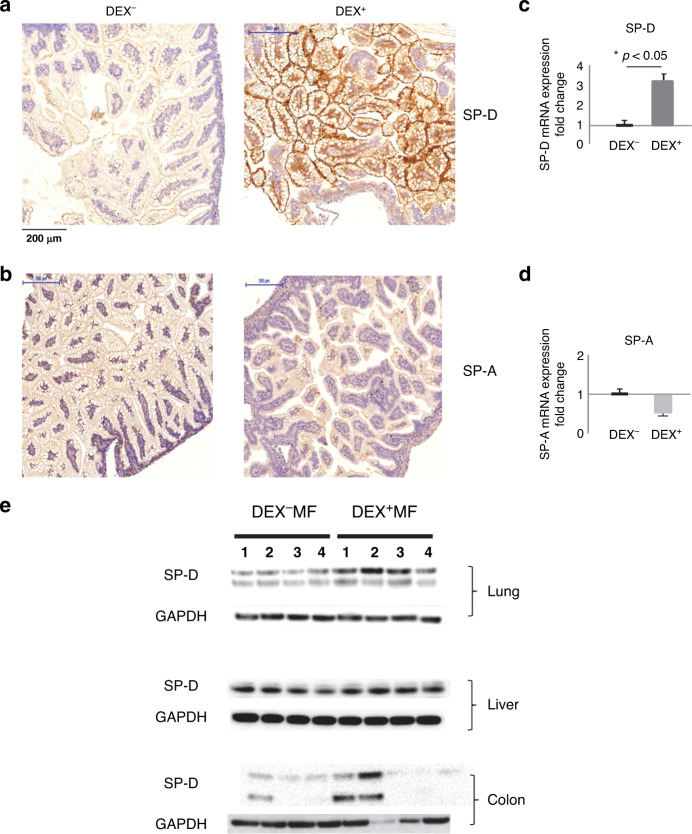
Antenatal dexamethasone affects the ileal mucosal surface expression of SP-D but not SP-A in the rat model of experimental NEC-like injury. Immunohistochemistry detection of SP-D localization in the neonatal ileum. Representative sections are shown. **A** PBS vs DEX. Antenatal dexamethasone increased SP-D expression on the ileal apical surface of DEX^+^FF pups compared to DEX^−^FF pups. Bar: 200 μM, magnification ×200. **B** DEX^−^FF vs DEX^+^FF. Immunohistochemistry detection of SP-A localization in neonatal NEC ileum. There is no detectable expression of SP-A on the neonatal rat ileal mucosal surface. **C** There is a significant upregulation of SP-D mRNA expression in ileal tissues of DEX^+^FF compared to DEX^−^FF pups *p* < 0.05 (*n* = 5 per group) **D** There is no detectable level of SP-A mRNA expression in DEX^−^FF and DEX^+^FF ileal tissues (*n* = 5 per group). **E** Western blots of SP-D expression in lung, liver and colon tissues of DEX^+^MF and DEX^−^MF pups (*n* = 4 per group).

SP-A and SP-D are involved in the essential innate immune response in protecting against pathogens.^27^ Since SP-A and SP-D have also been shown to protect against NEC in vivo and in vitro models,^17^ we explored the possibility that antenatal dexamethasone may modulate SP-A and/or SP-D expression resulting in the protection of the mucosa barrier in the neonatal rat ileum. As shown in Fig. 3a, dexamethasone induced a prominent expression of SP-D on the mucosal surface in response to experimental NEC challenge. In contrast, there was no detectable SP-A expression on the ileal mucosal surface (Fig. 3b). We also examined SP-D and SP-A gene expression in the rat ileum using RT-PCR. As shown in Fig. 3c, SP-D was expressed in the neonatal rat ileum and levels were elevated in DEX^+^FF pups when compared to the DEX^−^FF. There was no detectable SP-A expression in the rat ileum in our study (Fig. 3d). We also examined the SP-D expression in other organs in response to antenatal dexamethasone. As shown in Fig. 3e, DEX^+^MF pups expressed higher levels of SP-D in the lung and colon, while both DEX^−^MF and DEX^+^MF pup expressed comparable levels of SP-D by western blotting. In contrast, there were very low levels of either SP-D or SP-A mRNA expression in liver, colon, and ileum tissues in both groups by real-time RT-PCR testing. Unsurprisingly, we observed a high level of SP-D and SP-A gene expressions in the lungs but there is no significant difference between the groups (data not shown). We next focused our further investigation on intestinal SP-D and utilized an immature rat intestinal epithelial cell line (IEC-6) to elucidate the possible cellular mechanisms by which SP-D protects intestinal barrier function.

### SP-D protects IEC-6 cells during PAF/LPS challenge and preserves intestinal cell surface localization of TJ protein occludin

**Fig. 4:**
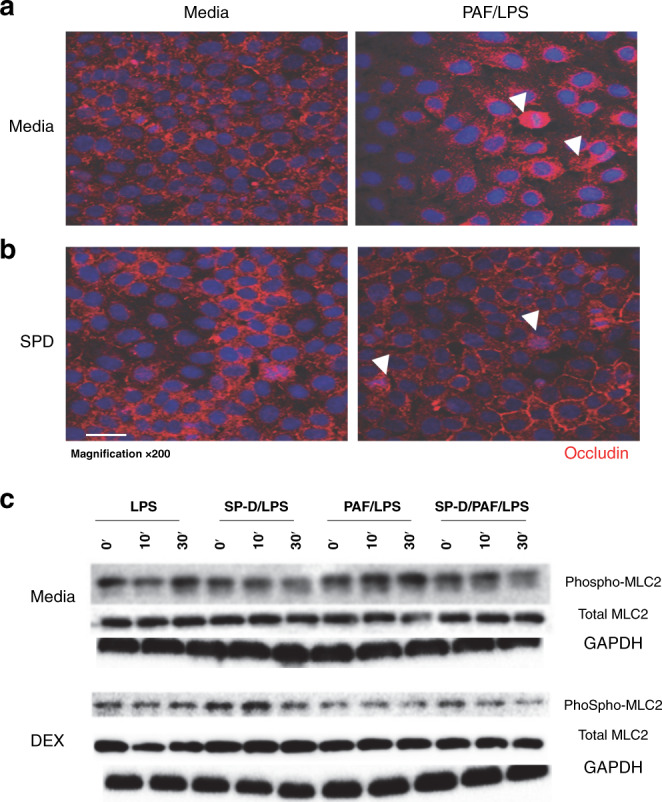
SP-D protects tight junction integrity in IEC-6 cells during PAF/LPS-mediated injury. SP-D pretreatment preserved cell surface OCLN expression. **A** Representative images of immunofluorescence detection of OCLN (red) in IEC-6 cells grown on coverslips. (left panel) media alone and (right panel) PAF/LPS. PAF/LPS challenge resulted in the loss of cell surface OCLN expression and cell–cell contact (block arrow). **B** IEC-6 cells pretreated with SP-D: left panel, SP-D/media and right panel, SP-D/PAF/LPS. Pretreatment of SP-D-protected IEC-6 cell–cell contact and cell surface OCLN expression (indicated by block arrows). **C** phosphorylation of MLC2 in the presence of SP-D (upper panel) and/or dexamethasone (lower panel) in IEC-6 cells during PAF/LPS challenge. LPS and PAF/LPS stimulation-induced phosphorylation of MCL2 at 30 min. SP-D alone can inhibit this response. Pretreatment with dexamethasone for 48 h enhanced SP-D inhibition of MCL2 phosphorylation. Western blotting of GAPDH was used as a control for equal loading.

PAF in combination with LPS has been established in clinical studies and animal models as important mediators of the pathophysiology of NEC.^28,29^ Thus, we challenged IEC-6 cells with PAF in combination with LPS (PAF/LPS) as a model to understand the cellular mechanism by which SP-D protects intestinal barrier function. We first investigated whether pretreatment of SP-D can preserve the integrity of TJ molecules on the cell surface in IEC-6 cells challenged with PAF/LPS. As shown in Fig. 4, pretreatment of SP-D exhibited protective effects on TJ protein OCLN (red). In IEC-6 cells, PAF/LPS exposure induced loss of cell–cell contact and massive intracellular accumulation of OCLN (Fig. 4a, right). In contrast, pretreatment of SP-D largely preserved cell surface OCLN expression and cell–cell contact (Fig. 4b, right). To investigate potential mechanisms by which SP-D preserve TJ integrity, we examined the signaling pathways that are implicated in the disruption of OCLN surface expression. Recent studies indicated the role of myosin light-chain kinase activation and myosin light-chain (MLC) phosphorylation mediates intestinal barrier dysfunction via OCLN endocytosis in various injury model.^30^ We examined phosphorylation of MLC2 in the presence of SP-D and/or dexamethasone in IEC-6 cells during PAF/LPS challenge. As shown in Fig. 4c, in the media control cells, PAF/LPS stimulation-induced phosphorylation of MCL2 at 30 min. SP-D alone can inhibit this response. When the cells were pretreated with dexamethasone for 48 h before SP-D exposure, there is strong inhibition of MCL2 phosphorylation induced by PAF/LPS stimulation.

### SP-D protects TJ assembly by promoting association between ZO-1 and APG2 at the cell membrane

**Fig. 5:**
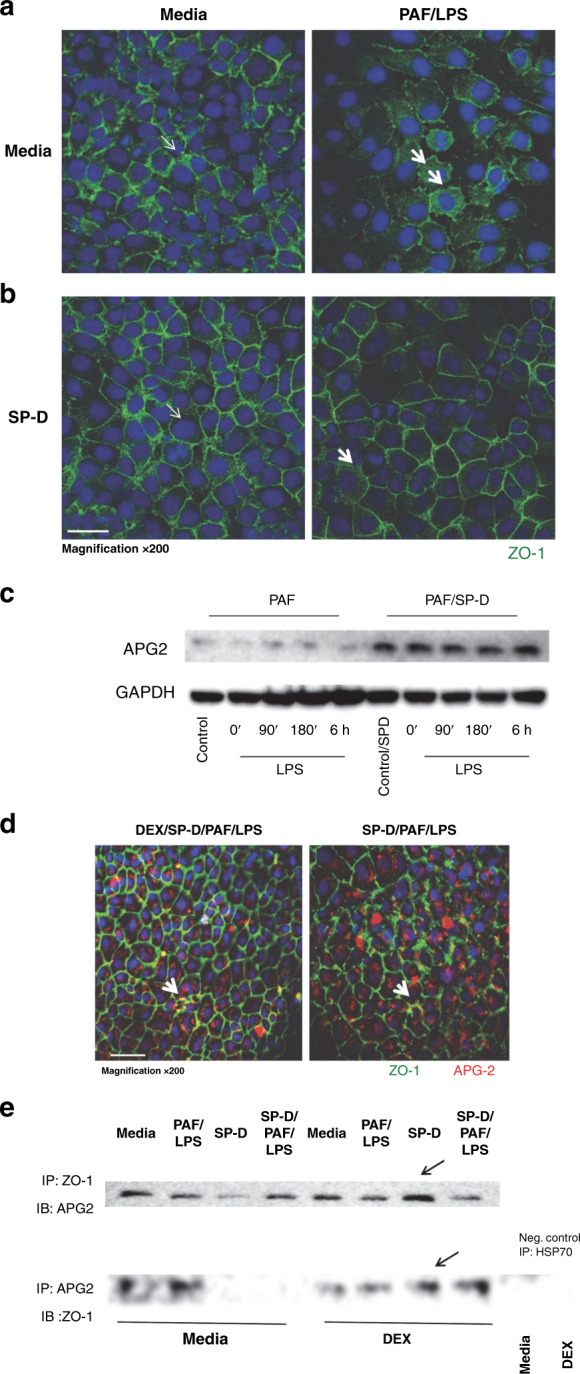
SP-D protects tight junction assembly by promoting association between ZO-1 and APG2 at the cell membrane. Representative images of immunofluorescence detection of ZO-1 molecules (green) on IEC-6 cells grown on coverslips: **A** (left panel) media alone and (right panel) PAF/LPS. PAF/LPS disrupted surface ZO-1 expression and cell–cell contact. **B** IEC-6 cells pretreated with SP-D: (left panel) SP-D/media and (right panel) SP-D/PAF/LPS. Pretreatment of SP-D-protected IEC-6 cell–cell contact and cell surface ZO-1 expression (indicated by block arrows). **C** Western blot of APG2 expression in IEC-6 cells. Pretreatment of SP-D can upregulate and preserve APG2 expression in IE-6 cells upon PAF/LPS challenge detected by Western blot. **D** Immunofluorescent staining of ZO-1 (green) and APG2 (red) on IEC-6 cell membrane. SP-D treatment preserve the association of ZO-1 and APG2 (colocalization shown with white arrow). Pretreatment with dexamethasone facilitated interaction between ZO-1 and APG2 at the cell surface. **E** Co-IP detection of ZO-1 and APG2 interaction in IEC-6 cells. Upper lane: using ZO-1 to pull down APG2; lower lane: using APG2 as bait to pull down ZO-1.

We next investigated whether pretreatment of SP-D can preserve the integrity of TJ molecules on the cell surface in IEC-6 cells challenged with PAF/LPS. As shown in Fig. 5a, PAF/LPS stimulation induced intracellular translocation of ZO-1 from the cell surface in IEC-6 cells resulting in the loss of the cell–cell contact that was evident in the media control cells. In contrast, SP-D preserved cell surface expression of ZO-1 and maintained cell–cell contact in the majority of the IEC-6 cells (Fig. 5b). There has been evidence suggesting that APG2, a chaperon protein that belongs to the HSP70 family, can interact with ZO-1 and play an important role in maintaining enterocyte polarity and TJ stability at the mucosal surface.^22^ Using western blot analysis, we show here that pretreatment with SP-D upregulates and preserves APG2 expression in intestinal epithelial cells during PAF/LPS challenge (Fig. 5c). In order to illustrate whether there is the direct interaction between ZO-1 and APG2, we first demonstrate that there is colocalization of ZO-1 and APG2 in IEC cells, and this interaction was strengthened when IEC-6 cells exposed to dexamethasone and SP-D as illustrated in Fig. 5d, white arrows indicate colocalization of ZO-1 (green) and APG2 (red) at the cell surface. Using the co-IP method, we were able to pull down either APG2 or ZO-1 when using the other protein as the bait, and furthermore, dexamethasone and/or SP-D may be able to strengthen the interaction as depicted in Fig. 5e.

## Discussion

NEC is a devastating gastrointestinal morbidity unique to premature infants. Little has changed in the treatment or outcomes since it was first described some 50 years ago.^31^ Since prematurity is the most important risk factor associated with NEC, studies using trophic agents, such as transforming growth factor-β, that protects murine pups against NEC-like injury by suppressing macrophage cytokine production and inhibiting the mucosal inflammatory responses; epidermal growth factor that reduces enterocyte apoptosis, and downregulates the inflammatory response associated with NEC-like injury; and corticosteroids that promote maturation of the gastrointestinal mucosal barrier have been explored as possible therapeutic approaches.^24,32–34^ However, what elements of maturation induced by corticosteroids critical to prevent NEC are poorly defined.

Antenatal corticosteroids are routinely used to ameliorate surfactant-deficient RDS that are associated with lung immaturity in premature infants.^8^ Studies have shown that in the fetal lungs, GCs stimulate the production of surfactant-associated proteins.^15^ There are four surfactant proteins, namely, SP-A, SP-B, SP-C, and SP-D.^35^ SP-B and SP-C proteins are only expressed in the pulmonary system and are involved in helping the spreading of surfactant across the alveolar lining and are also involved in surfactant metabolism and recycling.^36^ In the premature neonate, these proteins are important after birth in the susceptibility of pulmonary dysfunction resulting in RDS.^36^ SP-A and SP-D, on the other hand, are collectins with important roles in the maintenance of the pulmonary immune system and help in the first line of defense against various pathogens in the respiratory tract.^37^ Since then, alterations in SP-A and SP-D structure and function have been implicated in a wide variety of pulmonary diseases. In addition, over the past three decades, these proteins have become increasingly apparent in other organs, such as gastrointestinal tract. Both in vivo and in vitro experiments have demonstrated that SP-A and SP-D have protective effects against devastating NEC disease by downregulating Toll-like receptor 4-mediated inflammatory responses and protect mucosal barrier integrity.^19,38^

Antenatal corticosteroids therapy has also been shown to decrease the incidence of NEC and accelerate the development of the immature intestinal barrier in preterm infants. For instance, antenatal cortisone administration in neonatal rats during experimental NEC challenge lowers intestinal permeability and decreases translocation of macromolecules in the small intestinal mucosa of neonatal rats associated with reduced incidence and severity of NEC.^13,14^ However, very little is known about the cellular and molecular mechanisms. The present study adds to the current literature by demonstrating that the antenatal administration of the synthetic GC dexamethasone suppresses production of the inflammatory cytokines IL-1α, IL-β, and TNF-α, promotes intestinal TJ formation and prevents excessive destruction of TJ structures on the mucosal surface, leading to protection against intestinal injury in experimental NEC in neonatal rats.

Using an immature rat intestinal epithelial line IEC-6, we show that pretreatment with SP-D can specifically preserve the apical surface expression of TJ-associated proteins OCLD and ZO-1. Lastly, SP-D upregulated expression of a heat-shock protein APG2. Given the role of APG2 protein in regulating TJ assembly and epithelial cell differentiation via its interaction with ZO-1 molecules,^21,39^ this is a potential mechanism by which SP-D aids in the protection of mucosal barrier function.

Our data show that a single dose of antenatal corticosteroids can significantly decrease experimental NEC in a neonatal rat model. Antenatal dexamethasone is able to reduce inflammatory cytokine production and maintain intestinal barrier function. These protective effects might be mediated by its effect on the induction of levels of SP-D on the neonatal intestinal epithelial surface. However, one of the limitations of our study is the lack of functional in vivo study to determine if there is an association between the level of SP-D and pathophysiology of NEC in clinical patients or to elucidate the molecular mechanism by which SP-D can protect hosts against experimental NEC injury in human and animal studies.
